# Electroacupuncture Ameliorates Scopolamine‐Induced Dry Eye Disease in Mice by Restoring Lacrimal Gland Lipid Homeostasis and Reducing IL‐1β/IL‐18 Expression

**DOI:** 10.1155/jimr/5191118

**Published:** 2026-08-16

**Authors:** Shangjie Liang, Tengyan Ji, Xia Wu, Yunchuan Wu, Qingbo Wei, Ning Ding

**Affiliations:** ^1^ Nanjing University of Chinese Medicine School of Acupuncture-Moxibustion and Tuina and School of Health Preservation and Rehabilitation, Nanjing 210023, Jiangsu, China; ^2^ The Second Affiliated Hospital of Nanjing University of Chinese Medicine, Nanjing 210017, Jiangsu, China, njucm.edu.cn

## Abstract

Dry eye disease (DED) can lead to severe eye discomfort, which manifests as dryness, pain and decreased vision, and significantly affects daily life. This study elucidated the effects of electroacupuncture (EA) intervention on DED and clarified its potential mechanisms through changes in lacrimal gland lipid metabolism and inflammation. A DED mouse model was established by repeated subcutaneous injections of scopolamine hydrobromide, followed by EA intervention. The therapeutic effects of EA intervention on DED were determined based on tear secretion volume (*n* = 10), corneal fluorescein staining scores (*n* = 10), corneal stromal (CS) damage (*n* = 6), and changes in corneal and lacrimal gland morphology (*n* = 10). Mechanistic endpoints included lacrimal gland lipidomic profiling by ultra‐high‐performance liquid chromatography‐tandem mass spectrometry (UHPLC‐MS/MS) (*n* = 6) and assessment of IL‐1β and IL‐18 expression by immunohistochemistry (*n* = 6), western blotting (*n* = 3) and qRT‐PCR (*n* = 6). EA increased tear secretion, reduced corneal fluorescein staining and ameliorated CS and lacrimal gland pathological alterations. Lipidomic analysis identified 81 metabolites, and Kyoto Encyclopaedia of Genes and Genomes (KEGG) enrichment analysis highlighted arachidonic acid metabolism and inflammation‐related pathways. EA reduced arachidonic acid and several downstream pro‐inflammatory metabolites while partially restoring omega‐3 polyunsaturated fatty acids (PUFAs) and their derivatives. EA also reduced lacrimal gland IL‐1β and IL‐18 expression at the protein and mRNA levels. These findings suggest that EA may ameliorate scopolamine‐induced DED, at least in part, through modulation of lacrimal gland lipid homeostasis and suppression of inflammatory responses.

## 1. Introduction

Dry eye disease (DED) is a chronic ocular surface disorder characterised by a reduction in tear quantity or quality, primarily induced by inflammation [1–3]. In the early stages, DED primarily manifests as xerophthalmia, ocular swelling, photophobia, ophthalmalgia and visual fluctuations [4]. It may also progress to corneal epithelial (CE) defects, ulcers, scleritis and even irreversible visual impairment [5, 6]. The lacrimal glands are the core organs responsible for the production and secretion of the aqueous component of the tear film, which lubricates and moistens the ocular surface. Inflammation of the lacrimal glands leads to reduced tear secretion on the ocular surface and subsequent inflammation, which is one of the primary aetiologies of DED [7, 8]. Polyunsaturated fatty acids (PUFAs) are essential nutrients for the human body that participate in various physiological functions and exert a direct regulatory influence on immune responses [9–11]. Studies have shown that patients with DED often experience lipid metabolism disorders that lead to abnormal PUFA levels. This is characterised by an abnormal increase in omega‐6 PUFAs, resulting in tissue inflammation, and a decrease in omega‐3 PUFAs, which weakens their anti‐inflammatory capacity [12]. Relevant evidence suggests that supplementation with omega‐3 PUFAs can effectively improve DED [13]. Animal studies have further demonstrated that, compared with normal mice, the levels of omega‐6 PUFAs in the lacrimal glands of DED mice are significantly elevated, while the levels of omega‐3 PUFAs are significantly reduced [14, 15]. Therefore, improving the lipid metabolism of the lacrimal gland tissue and alleviating lacrimal gland inflammation may represent a potentially effective strategy for treating DED.

Electroacupuncture (EA) is a therapeutic modality that integrates electrophysiological techniques with acupuncture to stimulate specific acupoints on the body and modulate physiological states [16]. Several clinical investigations have demonstrated that EA can enhance tear secretion, augment tear film stability and repair corneal damage, thus exhibiting notable efficacy in alleviating symptoms and ensuring safety in patients with DED [17]. Experimental research has revealed that EA can treat DED by mitigating inflammation, enhancing neural regulation, upregulating tear mucin expression, controlling cell death and inhibiting oxidative stress [18]. Although EA has shown therapeutic effects in DED, whether these effects are mediated through the restoration of lacrimal gland lipid metabolism and the suppression of inflammatory cytokines remains unclear. This study aimed to further explore the potential mechanisms underlying the therapeutic effects of EA in the treatment of DED in mice by examining the alterations in the quantities of PUFAs and inflammation in the lacrimal glands of DED mice.

## 2. Materials and Methods

### 2.1. Animals

Male C57BL/6J mice were obtained from Beijing Vital River Laboratory Animal Technology Co., Ltd. and housed at the Animal Experiment Center of Nanjing University of Chinese Medicine (Animal Qualification Certificate Number 110011230106592481). Before the experiment commenced, all mice underwent a 1‐week period of acclimatisation. The housing conditions were as follows: the room temperature was maintained at 20–26°C, the relative humidity ranged from 40% to 70%, a 12 h light/dark cycle was maintained, and the mice had free access to food and water. All animal experiments were conducted in accordance with the guidelines and ethical standards established by the Animal Ethics Committee of Nanjing University of Chinese Medicine (Ethics Number 202304A025).

### 2.2. Experimental Design

Thirty male C57BL/6J mice were randomly allocated to the control (Con), model (Mod), and EA groups using a random number table, with 10 mice in each group. Based on the team’s prior research, a subcutaneous injection of 200 μL of scopolamine hydrobromide (0.25 mg/mL; Chengdu Pufei De Biotech Co., Ltd., JOT‐10515, Chengdu, China) was administered at 8:00, 11:00, 14:00 and 18:00 for 21 consecutive days to establish the DED mouse model, followed by an additional 14‐day period of model maintenance. The Con group received an equivalent dose of physiological saline by subcutaneous injection at the same time points. Starting on the 22nd day of model preparation, the EA group underwent EA intervention (HANS200A, Nanjing, China) on the periphery of the bilateral Jingming (BL1) and Taiyang (EX‐HN5) acupoints, with direct needle insertion to a depth of 2 mm, using alternating sparse‐dense waves at frequencies of 2 and 20 Hz and a current intensity of 1 mA for 15 min once daily for 14 days. All animals were subjected to inhalation anaesthesia.

Tear secretion and corneal damage were measured in each group on days 1, 7, 14, 21, 28 and 35 after the intervention. On day 35, confocal microscopy was used to observe corneal microlesions. After the intervention, all mice were euthanised by cervical dislocation, and the cornea and lacrimal gland tissues were isolated for haematoxylin and eosin (H&E) staining and lipidomic assessment.

With the exception of EA intervention, all other experiments concealed group assignments and were conducted separately by two researchers, who calculated the final results.

### 2.3. Phenol Red Thread (PRT) Test

A PRT (Tianjin Jingming New Technology Development Co., Ltd., Tianjin, China) was inserted into the conjunctival sac at the outer one‐third of the lower eyelid of each mouse. The stained red thread was removed after 20 s, its length was measured, and the tear volume was determined based on the length of the red thread.

### 2.4. Corneal Fluorescein Staining Score

A corneal staining filter paper strip (Tianjin Jingming New Technology Development Co., Ltd., Tianjin, China) was moistened with saline and placed on the lower conjunctiva fornix of the mice. Blinking allowed sodium fluorescein to be distributed quickly and evenly across the surface of the mouse cornea. The corneal epithelium was stained with sodium fluorescein and evaluated using the slit lamp examination method combined with the cobalt‐blue grading method for scoring. According to the keratopathy classification, the cornea was divided into four quadrants. The scoring criteria for each quadrant were as follows: 0 points for no staining, 1 point for fewer than five staining spots, 2 points for five or more staining spots and 3 points for patchy or filamentous staining. The total score for each eye was obtained by summing the scores of the four quadrants, with a maximum possible score of 12 points.

### 2.5. In Vivo Confocal Microscopy (IVCM)

Following the fixation of the mouse head using a confocal corneal microscope (Heidelberg HRT III RCM, Heidelberg, Germany), 0.4% oxybuprocaine hydrochloride eye drops (Santen Pharmaceutical Co., Ltd., HJ20215002, Osaka, Japan) were used for topical anaesthesia. After the eyelids were fixed, a Vidisic transparent gel was applied to the surface of the 40x immersion objective lens, and subsequently, the objective lens was gradually advanced. Once the corneal endothelial cells were clearly centred within the display area, the recording button was pressed to preserve images of the diverse layers of the cornea.

### 2.6. HE Staining

After the intervention, the mice were euthanised, and the central portion of the cornea and lacrimal gland tissues was collected, fixed in 4% paraformaldehyde for 24 h, paraffin‐embedded, sectioned, dehydrated with gradient ethanol, stained with H&E and observed under a light microscope for morphological changes (U‐ND6‐2 Fluorescence Multifunctional Microscope, Olympus, Tokyo, Japan) in the cornea and lacrimal glands.

### 2.7. Lipid Extraction

After adding 300 μL of extraction solution (80% methanol/H_2_O, pre‐cooled at −40°C and containing an isotopically labelled internal standard mixture), the lacrimal gland tissues were vortexed for 30 s, homogenised at 35 Hz for 4 min and sonicated for 5 min in an ice‐water bath. Homogenisation and sonication cycles were performed twice. After incubation at −40°C for 1 h, the samples were centrifuged at 12,000 rpm at 4°C for 15 min. An aliquot of 240 μL of the supernatant was taken, followed by the addition of 160 μL of ultrapure water. The mixture was vortexed for 30 s and purified using solid‐phase extraction. Following purification, the eluent was dried under a nitrogen stream, re‐dissolved in 50 μL of 30% acetonitrile, vortexed for 30 s, homogenised at 60 Hz for 4 min and sonicated in an ice‐water bath for 5 min. After centrifugation at 12,000 rpm at 4°C for 1 min, the reconstituted solution was transferred to an Eppendorf (EP) tube equipped with a filter membrane. Finally, after centrifugation under the same conditions for 15 min, the clear supernatant was subjected to ultra‐high‐performance liquid chromatography‐tandem mass spectrometry (UHPLC‐MS/MS) analysis (Waters Corporation, Acquity UPLC H‐Class, Milford, USA).

### 2.8. UHPLC‐MS/MS Analysis

The chromatographic separation of the target compounds was conducted using an ACQUITY Premier UHPLC system. Mobile phase A consisted of 0.01% formic acid in water, whereas mobile phase B consisted of 0.01% formic acid in acetonitrile. The injection volume was set at 10 μL, with the column temperature maintained at 50°C and an autosampler temperature at 4°C. A SCIEX Triple Quad 6500+ mass spectrometer was employed for mass spectrometry analysis in the multiple reaction monitoring (MRM) mode. Flow injection analysis was used to optimise the MRM parameters for each target analyte. The ion pairs exhibiting optimal responses were selected for quantitative analysis, whereas the remaining ion pairs were used for qualitative analysis.

Following data preprocessing, 81 of the 130 targeted lipid metabolites were retained for statistical analysis. For each pairwise comparison, Student’s *t*‐test was used to calculate the raw *p* values, and fold change was calculated as the ratio of the mean metabolite abundance between groups. VIP values were obtained from the corresponding orthogonal partial least squares discriminant analysis (OPLS‐DA) models. Raw *p* values were adjusted across the 81 tested metabolites using the Benjamini–Hochberg false discovery rate procedure. Candidate differential metabolites were defined by VIP >1.0, fold change ≥1.2 or ≤0.833, and raw *p* < 0.05. The corresponding FDR‐adjusted *p* values were reported to assess robustness considered significant after multiple‐testing correction. Kyoto Encyclopaedia of Genes and Genomes (KEGG) enrichment analysis was performed separately for each comparison using only the candidate differential metabolites, and pathway‐level *p* values were corrected for multiple tests.

### 2.9. Immunohistochemistry

All lacrimal gland tissue sections were dewaxed and hydrated. Following antigen retrieval with citrate buffer, 3% hydrogen peroxide was used to inhibit endogenous peroxidase activity, and 5% bovine serum albumin was used to block non‐specific labelling. Primary antibodies against IL‐1β (1:200; Cat. Number ab283818, Abcam, Cambridgeshire, UK) and IL‐18 (1:200 dilution; Cat. Number ab207323, Abcam, Cambridgeshire, UK) were applied to the tissues, incubated at 4°C, and subsequently reacted with HRP‐conjugated secondary antibodies at room temperature for 1 h. Photographic documentation was performed after the DAB chromogenic reaction. Quantitative analysis was conducted using ImageJ software.

### 2.10. Western Blotting

The lacrimal gland tissue was lysed using a buffer containing protease and phosphatase inhibitors, homogenised and lysed on ice. After centrifugation, the supernatant was collected. Total protein was quantified using a bicinchoninic acid assay kit. Subsequently, the samples were mixed with 5× sodium dodecyl sulphate‐polyacrylamide gel electrophoresis (SDS‐PAGE) loading buffer and denatured at 100°C for 10 min. Protein samples were separated using 10% SDS‐PAGE to detect specific proteins. The target proteins were then transferred to a PVDF membrane and blocked with 5% non‐fat milk for 2 h. The PVDF membrane was then incubated overnight at 4°C with the corresponding primary antibodies IL‐1β (1:1000 dilution; Cat. Number ab283818; Abcam, Cambridge, UK), IL‐18 (1:1000 dilution; Cat. Number ab207323; Abcam, Cambridgeshire, UK) and β‐actin (1:5000 dilution; Cat. Number 66009‐1‐Ig; Proteintech, Rosemont, USA). The membrane was then incubated with secondary antibodies for 1 h. Finally, the membrane was developed using the ECL reagent (BL520B, Biosharp, Anhui, China), and the greyscale values were analysed using ImageJ software.

### 2.11. qRT‐PCR

RNA extraction was performed using an RNA extraction kit for lacrimal gland tissue RNA (BS258A; Biosharp, Anhui, China), followed by the assessment of RNA concentration and purity. After cDNA synthesis (11139ES60; Yeasen Biotechnology Co., Ltd., Shanghai, China), PCR amplification was conducted. The qRT‐PCR cycling conditions were as follows: an initial denaturation at 95°C for 5 min, followed by 40 cycles of denaturation at 95°C for 10 s, annealing at 60°C for 20 s and extension at 72°C for 20 s. Table 1 lists the specific primers used to detect the target gene sequences. The changes in gene expression were normalised against the internal Con gene‐β‐actin.

**Table 1:** Primer sequences for qRT‐PCR.

Gene		Primer sequence (5′–3′)	Product length (bp)
*IL-1β*	Forward	GCAACTGTTCCTGAACTCAACT	89
	Reverse	ATCTTTTGGGGTCCGTCAACT	
*IL-18*	Forward	GACTCTTGCGTCAACTTCAAGG	169
	Reverse	CAGGCTGTCTTTTGTCAACGA	
*β-actin*	Forward	GGCTGTATTCCCCTCCATCG	154
	Reverse	CCAGTTGGTAACAATGCCATGT	

### 2.12. Statistical Analyses

Data analysis was performed using statistical software SPSS 26.0. The measurement data were expressed as the mean ± standard deviation ($\overset{―}{X}\pm S$). When the data conformed to a normal distribution and homogeneity of variance, an independent sample *t*‐test was used for comparisons between two groups, whereas a one‐way ANOVA was used for comparisons among multiple groups. When the variances were unequal, the rank‐sum test was used. If the data did not follow a normal distribution, non‐parametric tests were used. Statistical significance was defined as *p* < 0.05.

## 3. Results

### 3.1. EA Intervention Increased Tear Volume in DED Mice

**Figure 1:**
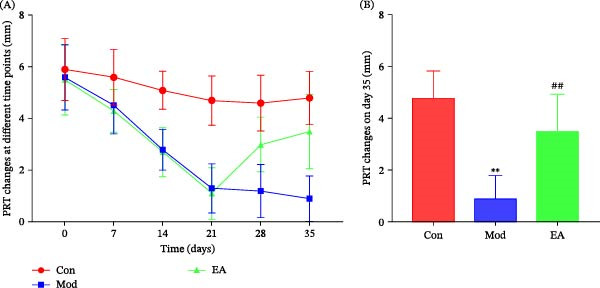
Effect of EA treatment on tear volume in DED mice. (A) Trend of tear volume changes before and after intervention. (B) Tear volume of each of the groups on the 35th day. *n* = 10 in each group. Compared with the Con group,  ^∗∗^ *p* < 0.01; compared with the Mod group, ^##^ *p* < 0.01.

DED typically manifests as inadequate tear secretion. The PRT test is a technique employed in clinical and experimental investigations to evaluate basal tear secretion function by quantifying the volume of tear secretion. As depicted in Figure 1, compared with the Con group, the Mod group exhibited a substantial reduction in tear secretion on the 21st day of model establishment, suggesting the successful construction of the DED model. After EA intervention, tear secretion in the mice was significantly increased.

### 3.2. EA Intervention Alleviated CE Damage in DED Mice

**Figure 2:**
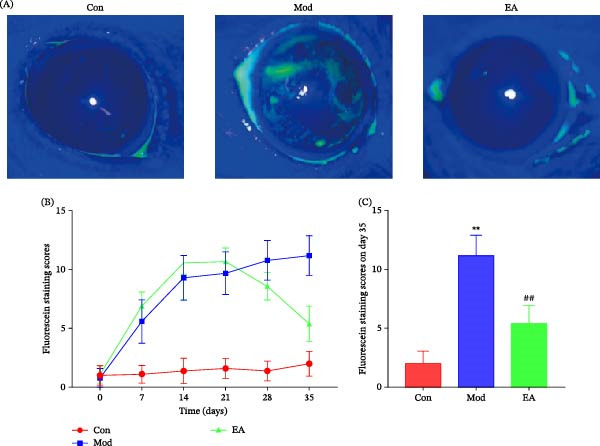
Effect of EA intervention on corneal epithelial damage in DED mice. (A) Fluorescent staining pictures of the cornea. (B) Trend of changes in fluorescein staining before and after intervention. (C) Fluorescein staining score of each group on the 35th day. *n* = 10 in each group. Compared with the Con group,  ^∗∗^ *p* < 0.01; compared with the Mod group, ^##^ *p* < 0.01.

The integrity of the corneal epithelium was evaluated using sodium fluorescein staining to assess ocular surface damage. As depicted in Figure 2A, subsequent to the intervention, no significant staining of the corneal epithelium was detected in the Con group. In contrast, the Mod group showed CE staining, which manifested as patchy, filamentous and branching patterns. The EA group exhibited slight punctate staining of the corneal epithelium. Moreover, quantitative scoring based on corneal staining revealed that, compared with the Con group, the corneal sodium fluorescein staining score in the Mod group significantly increased on the 21st day of model establishment, and no improvement was observed on the 35th day. Compared with the Mod group, the EA group showed a significant reduction in the corneal sodium fluorescein staining score on the 35th day (Figure 2B,C).

### 3.3. EA Intervention Alleviated Stromal Inflammation in the Corneas of DED Mice

**Figure 3:**
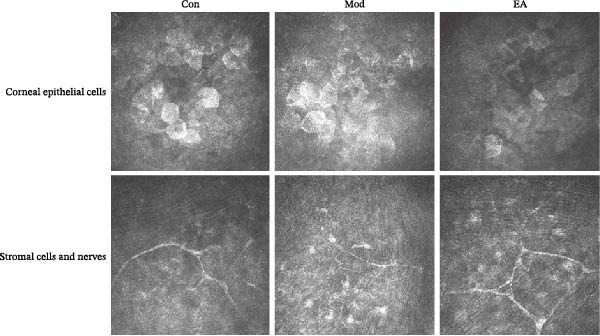
Effect of EA intervention on CE, CS and CSN in DED mice. The IVCM images of the anterior and posterior stromal layers under the cornea on the 35th day (800x). *n* = 6 in each group.

As depicted in Figure 3, IVCM was used to examine corneal microstructural changes throughout the corneal tissue, with representative images focusing on CE cells, corneal stromal (CS) cells and CS nerves (CSN). In the Con group, CE were uniformly distributed and structurally intact, with clearly defined cell contours and regular morphology. The stromal cells exhibited clear boundaries and a relatively uniform distribution, with intact and well‐organised stromal nerve fibres. In contrast, the Mod group showed pathological alterations, characterised by a disordered arrangement of CE, blurred cell boundaries, decreased overall imaging signal intensity, increased numbers of highly reflective CS, blurred cellular outlines and irregular cellular distribution. Additionally, CSN fibres in the Mod group appeared thinner, fragmented and exhibited reduced continuity and branching, indicating significant corneal nerve damage. Notably, EA treatment markedly reversed the pathological changes. Compared with the Mod group, the EA group showed a more regular arrangement of CE, clearer cell boundaries and restored overall structural continuity. Meanwhile, CS hyper‐reflectivity was reduced, and the cellular arrangement was more homogeneous. Moreover, CSN fibres exhibited improved continuity and partially restored branching patterns, suggesting a protective or regenerative effect of EA.

### 3.4. EA Intervention Improved the Corneal and Lacrimal Gland Tissue Morphology in DED Mice

**Figure 4:**
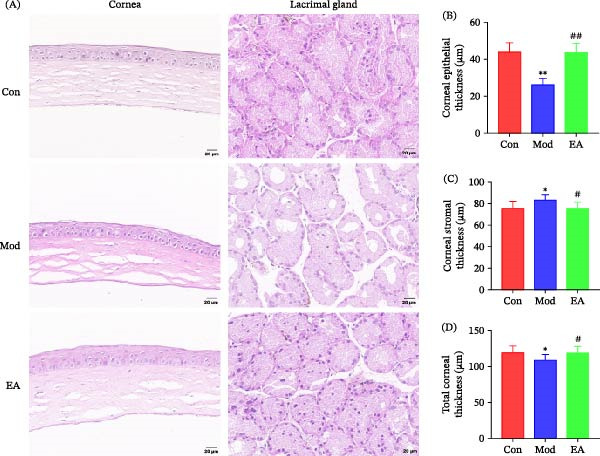
Effect of EA intervention on the morphological structure of the cornea and lacrimal gland in DED mice. H&E staining pictures of the cornea and lacrimal gland. *n* = 10 in each group. (A) H&E staining pictures of the cornea and lacrimal gland. (B) Corneal epithelial thickness. (C) Corneal stromal thickness. (D) Total corneal thickness. *n* = 10 in each group. Compared with the Con group,  ^∗∗^ *p* < 0.01,  ^∗^ *p* < 0.05; compared with the Mod group, ^##^ *p* < 0.01, ^#^ *p* < 0.05.

H&E staining was used to observe morphological alterations in the corneal and lacrimal gland tissues of each group of mice. As depicted in Figure 4A–D, the corneal and lacrimal gland tissue structures in the Con group were generally normal, with clear layers, moderate thickness and a well‐organised cell arrangement. The stromal collagen layers were dense with minimal gaps, and no significant inflammatory cell infiltration was observed. The lacrimal gland acini were tightly arranged with clear contours, the cytoplasm appeared relatively full and no obvious interstitial separation was observed. In the Mod group, significant histological damage was observed. The thickness of the corneal epithelium was reduced, accompanied by an irregular arrangement Figure 4B. The stromal layer exhibited increased loosening and gap‐like changes, accompanied by thickening, suggesting aggravated stromal oedema and collagen lamellar separation (Figure 4C). Scattered inflammatory cells were observed locally. The lacrimal gland demonstrated degenerative changes in the acini (faint staining or vacuolar changes), with more pronounced acinar/ductal dilation and enlarged interstitial gaps between the acini, suggesting damage to the secretory units and an interstitial response. The EA group showed a trend towards improvement compared with the Mod group. The corneal epithelium became more organised, with increased thickness. Stromal loosening and gap‐like changes were reduced, and the overall morphology more closely resembled that of the Con group, although mild residual oedema‐like changes were still visible. The lacrimal gland acini became fuller and more tightly arranged, with reduced cystic dilation, although focal areas of dense cell infiltration were still observed in some fields, suggesting the possible presence of mild residual inflammatory infiltration or repair responses.

### 3.5. EA Intervention Can Regulate the Lipid Metabolism of the Lacrimal Gland in DED Mice

**Figure 5:**
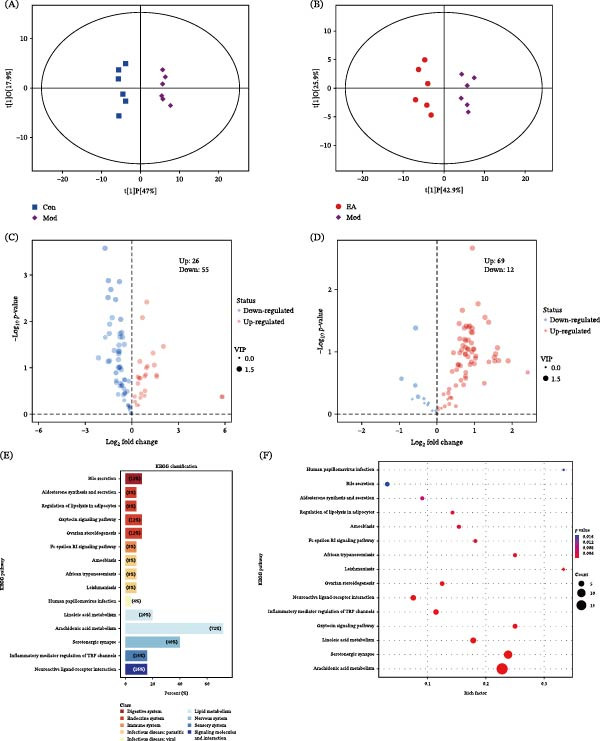
EA intervention can regulate the lipid metabolism of the lacrimal gland in DED mice. (A) OPLS‐DA between the Con and Mod groups. (B) OPLS‐DA between the Mod and EA groups. (C) Volcano plot between the Con and Mod groups. (D) Volcano plot between the Mod and EA groups. (E) Classification of the 81 significantly altered metabolites in all groups. (F) KEGG pathway enrichment of the 81 metabolites. *n* = 6 in each group.

The lacrimal glands exhibit a high lipid content, and the onset and amelioration of DED are intricately associated with the lacrimal gland function, which is typically accompanied by alterations in lipid metabolism. Consequently, we performed non‐targeted lipidomic analysis on the lacrimal gland tissues of each group of mice using UHPLC‐MS/MS. As depicted in Figure 5A,B, OPLS‐DA and principal component analysis (PCA) demonstrated substantial metabolic disparities between the Con group and Mod groups and between the Mod and EA groups. Following metabolite identification and quantification (Figure 5C,D), 81 differential metabolites were identified. Compared with the Con group, the levels of 26 metabolites were elevated in the lacrimal glands of the Mod group, whereas those of 55 metabolites were reduced. Compared with the Mod group, the levels of 69 metabolites were increased in the lacrimal glands of the EA group, whereas the levels of 12 metabolites were decreased. Figure 5E shows that the metabolites related to lipid metabolism accounted for a predominant proportion of the 81 differential metabolites. KEGG enrichment analysis indicated significant enrichment of arachidonic acid (ARA) metabolism (Figure 5F).

### 3.6. EA Intervention Reduced the Levels of Arachidonic Acid and Its Metabolites in the Lacrimal Gland

**Figure 6:**
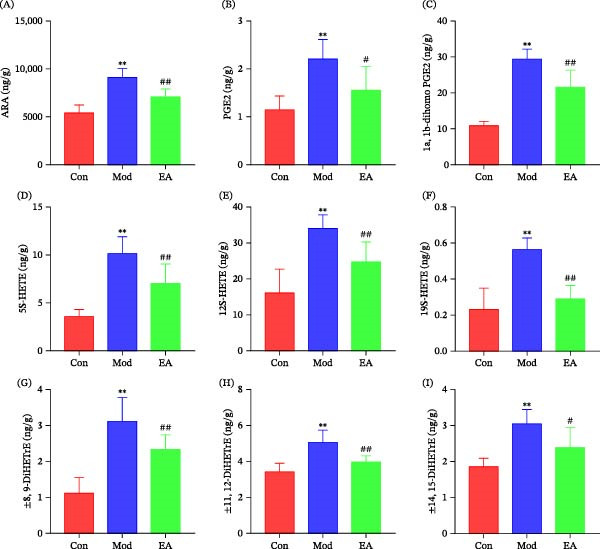
The effect of EA intervention on arachidonic acid metabolism in lacrimal gland tissue. (A) ARA. (B) PGE2. (C) 1a,1b‐dihomo PGE2. (D) 5S‐HETE. (E) 12S‐HETE. (F) 19S‐HETE. (G) ±8,9‐DiHETrE. (H) ±11,12‐DiHETrE. (I) ±14,15‐DiHETrE. *n* = 6 in each group. Compared with the Con group,  ^∗∗^ *p* < 0.01; compared with the Mod group, ^##^ *p* < 0.01, ^#^ *p* < 0.05.

As depicted in Figure 6, compared with the Con group, the ARA content in the lacrimal gland tissue of the Mod group exhibited a significant increase. Concurrently, the levels of its metabolites, PGE2 1a, 1b‐dihomoo PGE2, 5S‐hydroxyeicosatetraenoic acid (5S‐HETE), 12S‐hydroxyeicosatetraenoic acid (12S‐HETE), 19S‐hydroxyeicosatetraenoic acid (19S‐HETE), ±8,9‐dihydroxyeicosatrienoic acid (±8,9‐DiHETrE), ±11,12‐dihydroxyeicosatrienoic acid (±11,12‐DiHETrE) and ±14,15‐dihydroxyeicosatrienoic acid (±14,15‐DiHETrE), also significantly increased (all *p* < 0.01; Figure 6A–I). In contrast, compared with the Mod group, the levels of ARA and its metabolites in the lacrimal gland tissue of the EA group were significantly decreased.

### 3.7. EA Intervention Improved the Metabolic Disorder of Omega‐3 PUFAs in the Lacrimal Gland Tissue

**Figure 7:**
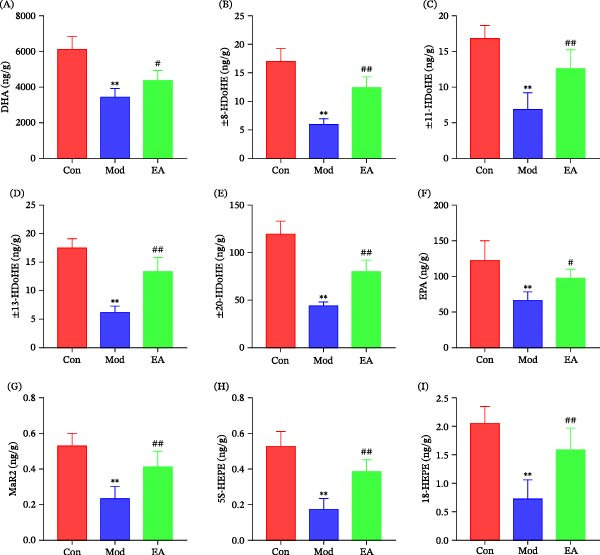
The effect of EA intervention on omega‐3 PUFAs and their metabolites in lacrimal gland tissue. (A) DHA. (B) ±8‐HDoHE. (C) ±11‐HDoHE. (D) ±13‐HDoHE. (E) ±20‐HDoHE. (F) EPA. (G) MaR2. (H) 5S‐HEPE. (I) 18‐HEPE. *n* = 6 in each group. Compared with the Con group,  ^∗∗^ *p* < 0.01; compared with the Mod group, ^##^ *p* < 0.01, ^#^ *p* < 0.05.

Normal metabolism of omega‐3 PUFAs is intricately associated with inflammatory homeostasis. Docosahexaenoic acid (DHA) and eicosapentaenoic acid (EPA) are the primary active constituents involved in anti‐inflammatory processes. As depicted in Figure 7, compared with the Con group, the Mod group exhibited a notable reduction in DHA and its metabolites. Conversely, the EA group mitigated this decrease compared with the Mod group. Similarly, compared with the Con group, the Mod group demonstrated a significant decline in EPA and its metabolites, and the EA group similarly attenuated this decline.

### 3.8. EA Intervention Alleviated Inflammation in the Lacrimal Gland Tissue of DED Mice

**Figure 8:**
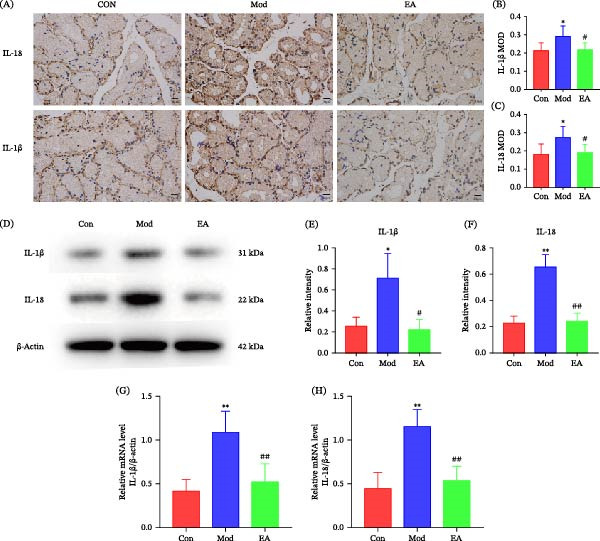
The effect of EA intervention on lacrimal gland tissue IL‐1β and IL‐18. (A) IHC staining of IL‐1β and IL‐18 in lacrimal gland tissue. (B, C) MOD values of IL‐1β and IL‐18; *n* = 6 in each group. (D) Western blotting analysis of each group. (E, F) Quantitative expression analysis of IL‐1β and IL‐18 in Western blotting; *n* = 3 in each group. (G, H) mRNA expression of IL‐1β and IL‐18 in lacrimal gland tissue; *n* = 6 in each group. Compared with the Con group,  ^∗^ *p* < 0.05,  ^∗∗^ *p* < 0.01; compared with the Mod group, ^#^ *p* < 0.05, ^##^ *p* < 0.01.

Inflammation is involved in lipid metabolism, and we further observed the effect of EA on lacrimal gland inflammation in mice with DED. IHC showed that, compared with the Con group, the expression of IL‐1β and IL‐18 in the Mod group was significantly increased, while the EA group significantly reduced their expression (Figure 8A). The quantitative results are represented by the MOD (Figure 8B,C). Western blotting was used to detect the protein expression levels of IL‐1β and IL‐18 in the lacrimal gland tissue, which showed that, compared with the Mod group, the EA group had reduced protein expression levels of IL‐1β and IL‐18 (Figure 8D–F). The mRNA expression levels of IL‐1β and IL‐18 detected by qRT‐PCR are shown in Figure 8G,H. Compared with the Mod group, the EA group had lower mRNA expression levels of IL‐1β and IL‐18.

## 4. Discussion

DED is one of the most common ocular surface diseases observed in clinical practice. Its incidence is increasing annually, attracting widespread global attention [19, 20]. Patients with DED often experience symptoms, such as dry eyes, pain and discomfort, which are associated with insufficient tear secretion [21–23]. Although the primary site of pathology in DED is clear, its pathogenesis is complex and remains incompletely understood. Research has shown that ocular surface damage caused by lacrimal gland inflammation is a key mechanism underlying DED [24]. Current DED treatments, such as sodium hyaluronate and corticosteroid eye drops, may provide short‐term relief but do not address the underlying disease. Moreover, long‐term use may cause adverse effects such as CE damage or increased intraocular pressure [25, 26]. Therefore, there is an urgent need to explore intervention strategies targeting lacrimal gland inflammation.

In DED, a marked disturbance occurs in lacrimal gland lipid metabolism, with increased pro‐inflammatory lipid metabolites that further exacerbate the inflammatory process. Thus, improving lipid metabolism and attenuating inflammation represent potential therapeutic strategies [27, 28]. EA, a modern medical therapy combining acupuncture and electrical stimulation, has demonstrated significant anti‐inflammatory effects in both clinical and basic research [29–31]. Additionally, previous studies have indicated that EA can significantly improve DED. Related studies have also shown that EA regulates lipid metabolism, although its role in DED and its relationship with lacrimal gland lipid metabolism and inflammation remain unclear [18, 32]. In this study, we established a DED mouse model to evaluate the effects of EA on DED and investigated its effects on lacrimal gland lipid metabolism and inflammation.

In clinical practice, patients with DED often experience varying degrees of ocular surface damage. Therefore, the international gold standard for diagnosing DED typically involves the assessment of tear secretion and ocular surface damage [33, 34]. In this study, compared with the Con group, mice in the Mod group exhibited significantly reduced tear secretion and CE damage, consistent with the basic characteristics of DED. Notably, after EA intervention, tear secretion in DED mice significantly increased and corneal damage was reduced. In recent years, IVCM has been used as a non‐invasive diagnostic tool to monitor morphological changes in the corneal layers and nerve distribution in real time, allowing objective assessment of ocular surface damage [35, 36]. In this study, compared with the Con group, the Mod group showed activated CS cells and an abnormal corneal nerve morphology. However, EA intervention effectively inhibited stromal cell activation and promoted the recovery of corneal nerve fibre density and branching. This suggests that EA exerts a dual repair effect on both ocular surface structure and function while effectively suppressing inflammation [37]. Furthermore, morphological examination of the cornea and lacrimal glands showed that EA effectively improved pathological damage in the lacrimal glands of DED mice, significantly reducing acinar atrophy and inflammatory infiltration. This indicates that EA has a clear therapeutic effect on DED by increasing tear secretion and reducing corneal and lacrimal gland damage, showing that the regression of lacrimal gland inflammation is an important indicator of therapeutic improvement [38].

Lacrimal glands are rich in lipids, which are among the most structurally diverse and functionally heterogeneous molecules in biological systems. Lipids play critical roles in processes such as cell membrane construction, energy metabolism, signal transduction and maintenance of cellular homeostasis [39–41]. PUFAs and their derived lipid mediators regulate the initiation, amplification and resolution of inflammation. In particular, lipid metabolic homeostasis in ocular surface tissues is important for maintaining tear film stability and suppressing excessive inflammation [42–46]. Relevant evidence indicates that DED is often accompanied by an imbalance in lacrimal gland PUFA metabolism, with an imbalance between omega‐6 and omega‐3 PUFAs exacerbating inflammation [47, 48]. In this study, multivariate statistical analyses (PCA/PLS‐DA) showed that the metabolic profiles of the Mod and Con groups exhibited clear separation, suggesting significant alterations in metabolic homeostasis under dry eye conditions. Volcano plots and KEGG pathway analysis further indicated that the differential metabolites were mainly enriched in pathways related to the inflammatory response, arachidonic acid metabolism, lipid signalling and immune‐related pathways, highlighting lacrimal gland inflammation as one of the core pathological mechanisms underlying DED. In the Mod group, various pro‐inflammatory lipid mediators and their metabolic precursors showed significant alterations, such as a marked increase in arachidonic acid (ARA) and its downstream metabolites (e.g., PGE2), whereas anti‐inflammatory or inflammation‐resolving PUFAs and their derivatives (e.g., DHA, EPA and MaR2) were significantly reduced. This ‘pro‐inflammatory enhancement–insufficient resolution of inflammation’ imbalance may exacerbate lacrimal gland and ocular surface inflammation through sustained activation of inflammatory signalling pathways, thereby promoting the onset and persistence of DED [49]. After EA intervention, the aforementioned lipid metabolism disorders significantly improved: ARA and PGE2 levels decreased, DHA, EPA and MaR2 levels increased, and pro‐inflammatory–anti‐inflammatory lipid mediators returned to normal. KEGG pathway enrichment analysis showed that EA modulated the arachidonic acid metabolism and inflammatory pathways. This not only provides a biochemical basis for its anti‐inflammatory effects but also supports the ‘lipid homeostasis regulation equals immune homeostasis regulation’ pathway for DED intervention.

This study suggests that the core mechanism by which EA improves DED may involve the regulation of inflammation resolution in the lacrimal gland. Studies have shown that the expression of inflammatory factors IL‐1β and IL‐18 in lacrimal glands is significantly elevated in DED, and inhibiting the expression of these inflammatory factors can effectively improve DED [50–52]. In this study, we observed that the transcriptional and protein expression levels of IL‐1β and IL‐18 in the lacrimal glands of the Mod group were significantly increased. This suggests that DED is associated with an inflammatory response, which is consistent with the previous morphological findings. Following EA treatment, the expression levels of these factors significantly decreased, indicating that the resolution of lacrimal gland inflammation is a key mechanism by which EA regulates DED. Although this study identified a close relationship between EA’s improvement in DED, lipid metabolism regulation and the resolution of inflammation, the upstream regulatory targets remain to be explored.

This study has some limitations. First, a sham‐EA or non‐acupoint stimulation group was not included; therefore, the nonspecific effects of handling, restraint, local needle insertion or electrical stimulation cannot be fully excluded. Second, only male C57BL/6J mice were used, and potential sex‐related differences in DED pathophysiology and EA responsiveness remain to be investigated. Furthermore, although animal allocation was performed using the random number table method, the implementation of blinding during the EA had limitations, which should be addressed in future studies. Third, the inflammatory analysis focused mainly on IL‐1β and IL‐18. Although these cytokines are closely related to inflammasome‐mediated inflammation, broader inflammatory profiling is needed [53]. Fourth, the western blot analysis included a relatively small number of biological replicates and should be interpreted together with the IHC and qRT‐PCR results. Finally, upstream pathway‐blocking experiments were not performed; therefore, the present data demonstrate associations among EA, lipid metabolic remodelling and reduced inflammatory cytokine expression but do not prove that these pathways are required for EA‐mediated protection. Future studies should include sham‐controlled designs, both male and female animals, larger validation cohorts and mechanistic investigations of pathways such as NLRP3 inflammasome activation and PUFA‐derived lipid mediator signalling.

## 5. Conclusion

This study confirms that EA may promote the active resolution of lacrimal gland inflammation by reshaping the lipid metabolism balance and inhibiting the expression of IL‐1β/IL‐18, thereby providing a promising therapeutic strategy for DED.

## Author Contributions

**Shangjie Liang**: data curation, formal analysis, writing – original draft, visualisation. **Tengyan Ji**: methodology, conceptualisation, visualisation, data curation. **Xia Wu**: investigation, software. **Yunchuan Wu**, **Qingbo Wei and Ning Ding**: writing – review and editing, funding acquisition, supervision, conceptualisation.

## Funding

This study was supported by the National Natural Science Foundation of China (Grants 82205259 and 82305377), Jiangsu Provincial Department of Science and Technology (Grant BK20230455), Nanjing Municipal Health Commission (Grant ZYYB 202229) and Jiangsu Education Department, Advantage Discipline of Traditional Chinese Medicine at Nanjing University of Chinese Medicine (Phase IV).

## Disclosure

All authors read and agreed to the published version of the manuscript.

## Ethics Statement

The animal study was approved by the Ethics Committee of Nanjing University of Chinese Medicine (Approval Number 202304A025). The study was conducted in accordance with the local legislation and institutional requirements.

## Conflicts of Interest

The authors declare no conflicts of interest.

## Data Availability

The data that support the findings of this study are available from the corresponding author upon reasonable request.
